# An assessment of the proportion of LGB+ persons in the Belgian population, their identification as sexual minority, mental health and experienced minority stress

**DOI:** 10.1186/s12889-022-14198-2

**Published:** 2022-09-23

**Authors:** Lotte De Schrijver, Elizaveta Fomenko, Barbara Krahé, Alexis Dewaele, Jonathan Harb, Erick Janssen, Joz Motmans, Kristien Roelens, Tom Vander Beken, Ines Keygnaert

**Affiliations:** 1 International Centre for Reproductive Health, Department of Public Health and Primary Care, Ghent University, Belgium, C. Heymanslaan 10, 9000 Ghent, Belgium; 2 Department of Psychology, University of Potsdam, Potsdam, Germany; 3 Department of Experimental Clinical and Health Psychology, Ghent University, Ghent, Belgium; 4 Institute for Family and Sexuality Studies, University of Leuven, Leuven, Belgium; 5 The Kinsey Institute, Indiana University, Bloomington, USA; 6 Transgender Infopunt, Ghent University Hospital, Ghent University, Ghent, Belgium; 7 Centre for Research on Culture and Gender, Ghent University, Ghent, Belgium; 8 Department of Obstetrics and Gynaecology, Ghent University Hospital – Ghent University, Ghent, Belgium; 9 Institute for International Research on Criminal Policy, Department of Criminology, Criminal Law and Social Law, Ghent University, Ghent, Belgium

**Keywords:** LGBT, Sexual orientation, Mental health, Minority health, Public health, We have no conflict of interest to disclose.

## Abstract

**Background:**

Previous studies report vast mental health problems in sexual minority people. Representative national proportion estimates on self-identifying LGB+ persons are missing in Belgium. Lacking data collection regarding sexual orientation in either census or governmental survey data limits our understanding of the true population sizes of different sexual orientation groups and their respective health outcomes. This study assessed the proportion of LGB+ and heterosexual persons in Belgium, LGB+ persons’ self-identification as sexual minority, mental health, and experienced minority stress.

**Method:**

A representative sample of 4632 individuals drawn from the Belgian National Register completed measures of sexual orientation, subjective minority status, and its importance for their identity as well as a range of mental-health measures.

**Results:**

LGB+ participants made up 10.02% of the total sample and 52.59% of LGB+ participants self-identified as sexual minority. Most sexual minority participants considered sexual minority characteristics important for their identity. LGB+ persons reported significantly worse mental health than heterosexual persons. Sexual minority participants did not report high levels of minority stress, but those who considered minority characteristics key for their identity reported higher levels of minority stress. LGB+ participants who did not identify as minority reported fewer persons they trust.

**Conclusions:**

The proportion of persons who identified as LGB+ was twice as large as the proportion of persons who identified as a minority based on their sexual orientation. LGB+ persons show poorer mental health compared to heterosexual persons. This difference was unrelated to minority stress, sociodemographic differences, minority identification, or the importance attached to minority characteristics.

**Supplementary Information:**

The online version contains supplementary material available at 10.1186/s12889-022-14198-2.

## Public significance statement

This study found that self-identified LGB+ persons make up at least 10% of the general population in Belgium, with only half of them identifying as sexual minority. Further, LGB+ persons experience worse mental health and well-being compared to heterosexual persons. Thus, it is important to further explore the risk and protective factors leading to health disparities, while recognizing the heterogeneous nature of this population and the importance of being sensitive to nuanced differences in subgroups within LGB+ populations. Measuring sexual orientation systematically in any population study is crucial to attain that goal.

## Background

Sexual minority people include people who are lesbian, gay, bisexual (LGB), pansexual, omnisexual, queer, questioning, fluid, asexual and have other sexual orientations [1], which we abbreviate as LGB+. LGB+ persons are considered a subgroup of the general population, or a sexual minority as their sexual identity, orientation, or practices differ from the majority of the society in which they live [2]. Yet, estimates of the proportion of people who belong to this subgroup are generally lacking since questions pertaining to sexual orientation are rarely integrated in representative population studies [3, 4]. In 2019, the Organisation for Economic Co-operation and Development (OECD) reported that in the 14 OECD countries where LGB+ estimates were available (i.e., Australia, Canada, Chile, France, Germany, Iceland, Ireland, Mexico, New Zealand, Norway, Sweden, United Kingdom, and the U.S.), 2.7% of the adult population identified as LGB [3]. For Belgium, national representative estimates of LGB+ persons are lacking. Yet, some representative regional estimates suggest that three to 8 % of the Flemish population identifies as LGB+ [5, 6]. For the Walloon region in Belgium, prevalence estimates are not available to our knowledge.

With this study, we want to contribute to the knowledge about the LGB+ persons in Belgium based on representative population data because the current lack of data regarding sexual orientation in population studies or census data limits our understanding of the size of the LGB+ population and their health outcomes [4, 7].

Although the available evidence is limited, Belgian studies based on convenience samples almost consistently show an association between identifying as LGB+ and negative mental health outcomes [8–11]. The evidence suggests that LGB+ persons are more at risk of developing certain mental disorders compared to heterosexual persons, such as depression, anxiety, suicide attempts or suicides, and substance-related problems [12–16]. Poorer health among LGB+ persons compared to heterosexual persons is most often explained by lifestyles and associated differences in sociodemographic situations [17–19] resulting in LGB+ persons showing more general risk factors for experiencing mental health problems (i.e., exposure to violence and abuse, sensation seeking, family factors, a lack of social support, financial difficulties etc.) [18–23]. Minority stress has been proposed to explain this observed increased risk [18, 24–27]. As such, studying minority stress is relevant to health outcomes research, particularly in studies regarding LGB+ persons. It refers to stress experienced as a result of one’s stigmatized social position by belonging to a minority. A person’s minority status can be the result of self-identification with a minority group as well as by appointment by others as a member of a minority group [24].

Minority stress theory describes the ways in which the everyday stress of living as a societal minority has a negative impact on the well-being [16, 28]. In addition to everyday stressors, distinct sexual minority experiences including victimization, prejudice and discrimination, negatively influence the well-being and health of this population disproportionately [16, 24]. Minority stress adds to general stressors, requiring an additional effort to cope with the stressful situation and should be considered as a chronic and socially based phenomenon since it is related to underlying social and cultural structures and processes beyond the individual level [24].

Minority stress emerges from three stress processes [24]. First, LGB+ persons experience distal objective external stressors which include all forms of structural or institutionalized discrimination and prejudice as well as direct interpersonal victimization experiences. These distal stressors occur independently of personal identification with the minority group. More centrally at play are processes involving anticipated social rejection or victimization which elicit vigilance related to these expectations. The third and most proximal process is the internalization of negative social attitudes, also known as internalized stigma/homophobia [16, 24, 29]. These processes are the most subjective since they rely on an individual’s perceptions and appraisals, and are related to self-identification as sexual minority. The concealment of one’s sexual identity can be seen as a proximal stressor since the associated stress effects are considered to stem from internal psychological processes. When something is central to one’s identity, being unable to safely express this part of oneself negatively affects a person’s well-being. Shaping and accepting an identity which is different from that of the dominant group and elicits shame and negative attributions, may result in internal conflicts. Accordingly, internalized stigma has repeatedly been linked to mental health problems [8, 13, 21, 24, 30, 31]. Intrapersonal psychological processes such as coping, emotion regulation and appraisals, mediate the link between experiences of minority stress and mental disorders [13, 16, 26, 32]. On the other hand, experiencing social support and positive social relations with both LGB+ and non-LGB+ persons has been identified as a potential protective factor [18, 21, 23, 24, 26, 33, 34].

Evidence regarding sexual minority mental health predominantly stems from data collected in student populations in the United States of America (USA). The Western-European cultural climate differs in terms of tolerance towards sexual and gender diversity [35, 36] and as such, the minority stress theory may potentially be less or differently applicable. First, because levels of minority stress experienced by Western-European LGB+ persons may be lower than experienced by American LGB+ persons as a result of more tolerant attitudes towards LGB+ persons in Western-Europe than in the USA, and secondly, because the pathways linking minority stress to mental health may be different. Yet, a national protective legal framework does not necessarily imply full social acceptance by civilians [37]. Although Belgium placed second on the Rainbow Index for the second time in a row in 2021 [36], LGB+ persons still experience ‘othering’ - a set of dynamics, processes, and structures which define and label some individuals or groups as not fitting in within the norms of a social group - and face stigma, prejudice and discrimination [38, 39]. Thus they may also experience minority stress and associated negative mental health outcomes.

### The current study

This study aimed to estimate the proportion of inhabitants of Belgium who self-identify as LGB+. In addition, we wanted to explore whether LGB+ individuals in our sample also identify as belonging to a sexual minority group in Belgium. Although LGB+ persons are often referred to as sexual minority people, this does not necessarily imply that LGB+ persons consider themselves to be part of a minority group in Belgium. Further, we wanted to study whether they experienced minority stress, and if their mental health outcomes vary depending on their self-identification as LGB+, as minority, and the importance for their identity they ascribe to their sexual orientation.

Our study had five specific objectives. First, we wanted to identify the proportion of persons who self-identify as LGB+ and as heterosexual in the Belgian population based on representative data (1). Second, we wanted to compare the observed mental health in LGB+ persons to that of heterosexual persons in our sample (2). We hypothesized that LGB+ identifying persons will report poorer mental health than heterosexual-identifying persons (Hypothesis 1).

Next, we focused on the proportion of LGB+ persons who also identify as belonging to a minority group in Belgium because of their sexual orientation (further referred to as ‘sexual minority’) (3) and examined whether they considered this minority status to be an important element for their identity (4). This resulted in three comparison groups: (a) those LGB+ participants who do not identify with a minority group related to their sexual orientation; (b) those LGB+ participants who do identify with a minority group related to their sexual orientation (sexual minority), but who do not consider this to be key for their identity; and (c) those LGB+ participants who do identify as sexual minority and who do consider this to be important for their identity. Based on this classification, we compared the observed mental health outcomes in these three groups (5) to test the hypothesis that LGB+ participants who identify as sexual minority and consider this characteristic as central to their identity, would show worse mental health outcomes than the other two LGB+ groups (Hypothesis 2).

## Method

### Sampling procedure and participants

Data were collected as part of a larger mixed-methods research project (‘UNderstanding the MEchanisms, NAture, MAgnitude and Impact of Sexual violence in Belgium’; UN-MENAMAIS) that included a cross-sectional online survey administered to a nationally representative sample of persons aged 16 to 69 years in Belgium. The Belgian National Register (BNR), containing demographic information (but not about sexual orientation) on all Belgian residents, served as the sampling frame for two periods of data collection. A random disproportionate stratified sample was drawn from the BNR with the aim to reach an equal number of male and female legal Belgian inhabitants equally divided into three age groups (i.e., 16–24 years old, 25–49 years old, and 50–69 years old). Overrepresentation of certain subgroups (e.g., male and female participants), was post hoc corrected using quota based sampling to obtain estimates representative of the population residing in Belgium (see [40] for more details).

The online survey was started by 6504 respondents. Respondents were excluded because they either did not give informed consent (*n* = 706), did not complete the survey (*n* = 909), did not meet criteria regarding age (i.e., between 16 and 69 years old; *n* = 6), completed the survey multiple times (*n* = 37), and because there were concerns about the quality of the responses (*n* = 1). Respondents who had missing values in key variables (e.g., items on sexual orientation) for this study were excluded as well (*n* = 213). The total final sample consisted of *n* = 4632, which corresponds to a response rate of 11.16%.

### Measures

#### Questionnaire development and validation

The UN-MENAMAIS survey included questions regarding sexual victimization and perpetration, but also questions on sociodemographic information, on sexuality and gender, mental health, quality of life and resilience, and minority identity which were analyzed for this paper. The initial version of the survey was developed in English by a multidisciplinary research consortium with a background in Health Sciences, Sociology, Psychology, Psychiatry, Criminology, Human Sexuality Studies, and Anthropology. Information about the generation and validation of all measures can be found elsewhere (see [40–42]).

The final version of the survey was translated into the three most commonly spoken languages in Belgium (i.e., Dutch, French, and English), and into Arabic, Farsi, and Pashtu which were at the time the three most spoken languages among refugees and applicants for international protection residing in Belgium (see [43]). The survey was completed 2886 times in Dutch, 1578 times in French, 154 times in English, nine times in Arabic and five times in Farsi. No one completed it in Pashtu.

#### Assessment of sex, gender, and sexual orientation

Following guidelines on collecting data on sexual orientation and gender identity [4, 44, 45], we used multiple-step questions to assess these variables. First, *sex* was measured by asking participants to name the sex they were assigned to at birth (male/female; the two only legal possibilities in Belgium). The second step entailed a multiple choice question “how do you describe yourself” allowing to answers as a man/as a woman/as transman/as transwoman/other, namely as …. . When participants chose the option “other, namely as”, they could write down their gender description of preference. Participants who self-identified as trans or other and participants who indicated a sex at birth different from their gender identity, were considered as non-cisgender participants. In this paper we compare findings based on the sex assigned at birth. Analysis based on gender identity falls beyond the scope of this study.

*Sexual orientation* was measured using multiple items: we asked participants to whom they felt sexually attracted, how they label their sexual orientation, and the gender of their sexual partners. This paper focuses on self-identifying LGB+ persons. The exploration of overlap between sexual attraction, self-labelling and sexual behavior is the focus of another study. To select the relevant subgroups in our sample, we asked to indicate which description applied to them: heterosexual; bisexual; gay/lesbian; pan−/omnisexual; asexual; other, namely …. The options pansexual and omnisexual were combined to limit the number of answer possibilities and the received feedback during the survey validation phase that both terms can be used as synonyms in our local context. Choosing “other, namely …” meant that they could complete their answer with their preferred sexual orientation label. Sexual orientation was recoded into a dummy variable LGB+/heterosexual. Hence, all participants who chose ‘heterosexual’ were labelled ‘heterosexual’. All others were grouped together into ‘LGB+’.

#### Assessment of minority identity

Participants were asked to indicate whether they considered themselves as belonging to a minority group in Belgium (yes/no) and if so, to indicate in a grid which characteristics (i.e., sexual orientation, gender identity, intersex or DSD condition, religion or life philosophy, skin color, ethnicity, disability, age or another characteristic) defined their minority status. Multiple answers were possible. In this study, we focused on LGB+ participants and their identification with a minority group based on sexual orientation related characteristics. The LGB+ participants were grouped in either the ‘sexual minority’ or the ‘non-sexual minority’ group.

Participants who indicated belonging to any minority group (e.g., sexual minority subgroup), received a binary follow-up question to assess the importance (i.e., important/not important) of each indicated characteristic for their identity.

#### Social support, substance use, mental health, and well-being

As a global measure of well-being, all participants were asked to rate their quality of life on a five-point Likert scale ranging from 1 = ‘very poor’ to 5 = ‘very good’. Specific mental health aspects were measured in all participants by validated scales from the international literature. *Depression* was assessed using the 9-item Patient Health Questionnaire (PHQ-9) [46]. Responses were made on a 4-point likert scale ranging from ‘not at all (0)’ to ‘nearly every day (3)’. All items were summed in a final score ranging from 0 to 27, Cronbach’s Alpha = .872. *Anxiety* was measured by the General Anxiety Disorder (GAD)-7 [47]. The scale had seven items, and responses were made on a four point likert scale ranging from ‘not at all (0)’ to ‘nearly every day (3)’, Cronbach’s Alpha = .890. All items were summed in a final score ranging from 0 to 21 to yield a total anxiety score. Both scales assessed symptoms in the 2 weeks prior to filling in the survey and both used a cut-off score of five as a positive screening for depression and/or anxiety [46, 47].

*Posttraumatic Stress Disorder (PTSD*) was measured using the PC-PTSD-5, which questioned symptoms in the month before the interview [48]. On this scale with five items with a response format of ‘yes (1)/no (0)’ answers, a score of three of a maximum of five was regarded as an indication for PTSD [48]. *Resilience* was assessed using the 6-item 5-point-Likert Brief Resilience Scale (BRS) (Cronbach’s Alpha = .814. All six items were averaged in a final score ranging from 0 to 5 [49].

*Hazardous alcohol* use was screened for using the AUDIT-C [50, 51]. The AUDIT-C consists of three questions, being ‘How often do you have a drink containing alcohol?’ ranging from ‘Never (0)’ to ‘4 or more times a week (4)’ (the screening ends with a score of 0 for respondents that indicated ‘Never’ in this first item), ‘How many standard drinks containing alcohol do you have on a typical day’ ranging from ‘1 or 2 (0)’ to ‘10 or more (4)’ and ‘How often do you have six or more drinks on one occasion?’ ranging from ‘Never (0)’ to ‘Daily or almost daily (4)’. In accordance to the guidelines of ‘Vlaamse Expertisecentrum voor Alcohol en andere Drugs (VAD)’, a cut-off score of four for females and five for males was used on this 3-item scale with a total score between zero and 12 [52]. In addition to the validated scales, participants were asked using yes-no questions about sedative use, cannabis use, illegal drug use, self-harm and suicide attempts, both during their lifetime and in the past 12-months. These questions were then combined into a variable per coping mechanism with categories ‘No (0)’, ‘Yes, during the lifetime, but not in the past 12-months (1) and ‘Yes, during the past 12 months (2)’.

*Social support* was assessed via four items analyzed as two variables. The first item inquired about with how many people one feels comfortable with to discuss secrets or private matters (i.e., variable: ‘number of trusted persons’). Every participant received this question and added the respective number in an open format. The three other items were only presented to those participants who indicated to belong to a minority group in Belgium because of their sexual orientation, gender identity, intersex or DSD condition, religion or life philosophy, skin color, and/or ethnicity. They received the Othering-Based Stress Scale (OBS-S) - which is an adapted version of the minority stress measure - relevant to the characteristic they had indicated. The OBS-S (see Additional file 1) was used to assess minority stress experienced in relation to either ‘sexual orientation and gender identity-related’ characteristics (i.e., sexual orientation and gender identity) or ‘cultural-related’ characteristics (i.e., religion or life philosophy, skin color, and/or ethnicity) and consisted of six subscales: identity concealment (3 items), micro-aggressions (3 items), rejection anticipation (3 items), victimization events (10 items), internalized stigma (3 items), and community connectedness (3 items). The community connectedness scale (i.e., the second variable) also served as a proxy to observe social support in these participants. Responses were made on a five-point scale ranging from ‘Strongly disagree (1)’ to ‘Strongly agree (5)’. The items from the last subscale community connectedness were rescaled from ‘Strongly disagree (5)’ to ‘Strongly agree (1)’ before creating a mean across all 25 items (Cronbach’s Alpha = 0.794) where ‘1’ equals ‘low othering-based stress’ and every value higher than four means high othering-based stress.

#### Ethical considerations and procedure

This study was approved by the Commission for Medical Ethics of Ghent University Hospital/Ghent University (B670201837542). It was designed and performed in line with the principles of the Declaration of Helsinki. This study only included participants of 16 years and older given ethical and practical regulations related to the legal age of consenting to sex, which is 16 years old in Belgium. All participants gave informed consent before initiating the online survey.

To limit self-selection bias, the study was presented as a broader survey about health, sexuality, and well-being. The sample size calculations based on the design of the UN-MENAMAIS study led to a required sample size of 5190 participants with a targeted 864 participants per subgroup. To reach this target while considering potential non-response and refusals to participate, four times the estimated required sample size was invited for participation (i.e., *N* = 41,520). Between 10/10/2019 and 01/01/2021 two independent waves of data collection took place. The second wave of data collection was meant to increase the sample size and quota based sampling was applied to balance the first wave of data collection and to reach a sufficient sample size per subgroup of interest. The sample comprised 2018 participants from the first wave and 2614 participants from the second wave of data collection.

The online survey was administered via the survey software Qualtrics (Qualtrics, Provo, UT, USA). Participants could access the self-administered survey using either a link or a Quick Response (QR) code, that could be scanned using a smartphone, as indicated in the letter sent by the BNR. Before participation, potential participants received online additional information on the study and an online informed consent form. Only upon informed consent were respondents able to proceed in the survey. To increase response rates, sampled potential participants received one reminder letter sent out again by the BNR 2 weeks after their initial invitation and all invitees were informed about the possibility to receive a raffled voucher worth 30 EUR upon participation. To take part in the latter, participants were directed to a separate short questionnaire after completing the main survey to ensure that survey answers could not be linked to personal contact information.

#### Analysis

All analysis were run in R4.1.1. Descriptive statistics (means, standard deviations, counts, and percentages) were computed for all variables figuring across all tables. Significant differences in the distribution of nominal variables between 1) participants who self-identified as heterosexual and participants who self-identified as LGB+, between 2) LGB+ participants who self-identified as being part of a minority group because of sexual orientation related characteristics (sexual minority) and LGB+ that did not self-identify as being part of a sexual minority group (Non-sexual-minority), as well as between 3) sexual minority participants who find their sexual orientation related characteristics important for their identity and sexual minority participants that do not find these characteristics important for their identity were computed using chi-square-tests. Chi^2^ tests going beyond 2 × 2 tables were followed up by post-hoc Chi^2^ tests to facilitate pairwise comparisons between categories. Effect sizes were explored by comparing the Cramer’s V coefficient (V). If the assumptions of a Chi^2^ test were not met, a Fisher’s Exact test was used. To compare the means of the continuous variables, the independent samples t-test was used. All assumptions were checked. The Levene’s Test was used to check for homogeneity of variance, which led to the use of the Welch t Test statistic if equal variances could not be assumed. Effect sizes were determined by calculating the Cohen’s d coefficient (D) if the sample size of the two groups were approximately the same or by using Hedges’ correction (G) if the sample size of the two groups were too different.

## Results

### Sample

The total sample consisted of 2300 male participants and 2332 female participants. The mean age of the sample was 39.07 years (SD = 17.02). In this sample, 4108 participants were born in Belgium. Out of those who were not born in Belgium, 231 persons held the Belgian nationality at the time of the survey. Further, 1020 persons had at least one parent who was not born in Belgium and 1316 persons had at least one grandparent who was not born in Belgium.

Table 1 summarizes the sociodemographic characteristics of the sample. In comparison to publicly available information on the level of education in the entire population, our sample appears to overrepresent higher educated people. Almost half of all respondents (i.e., 49.89%) completed a level of higher education, while - on the population level - 37.6% of Belgian residents between 15 and 64 years completed a higher educational level [53].

**Table 1:** Sample composition (*n* = 4632) & sociodemographic information

Variable	Within total sample (*n* = 4632)		X^2^; df; *p*-value; V	Within LGB+ group (*n* = 464)		X^2^; df; *p*-value; V
	Heterosexual (*n* = 4168; 89.98%) n (Valid %)	LGB+ (*n* = 464; 10.02%) n (Valid %)		Sexual Minority (*n* = 244; 52.59%) n (Valid %)	Non-sexual Minority (*n* = 220; 47.41) n (Valid %)	
**Sex assigned at birth**			2.27; 1; .132; .022			.539; 1; .463; .034
** Female**	2083 (49.98)	249 (53.66)		127 (52.05)	122 (55.45)	
** Male**	2085 (50.02)	215 (46.34)		117 (47.95)	98 (44.55)	
**Age groups*[mean (SD)]***	*39.68 (17.12)*	*33.63 (15.11)*	8.07; 603; <.001; .357*	*29.60 (13.07)*	*38.10 (15.96)*	6.30; 415; <.001; .585*
** 16–24 years old**	1254 (30.09)	198 (42.67)		133 (54.50)	65 (29.55)	
** 25–49 years old**	1374 (32.96)	174 (37.50)		86 (35.25)	88 (40.00)	
** 50–69 years old**	1540 (36.95)	92 (19.83)		25 (10.25)	67 (30.45)	
**Educational level**			10.44; 2; .005; .047			5.37; 2; .068; .108
** Primary education or none**	255 (6.12)	26 (5.60)		17 (6.97)	9 (4.09)	
** Secondary education**	1803 (43.26)	237 (51.08)		113 (46.31)	124 (56.36)	
** Higher education**	2110 (50.62)	201 (43.32)		114 (46.72)	87 (39.55)	
**Occupational status**			25.39; 2; <.001; .074			31.84; 2; <.001; .262
** Remunerated workforce**	2151 (51.61)	196 (42.24)		99 (40.57)	97 (44.09)	
** Student**	1034 (24.81)	164 (35.34)		111 (45.50)	53 (24.09)	
** Other**	983 (23.58)	104 (22.41)		34 (13.93)	70 (31.82)	
**Financial situation**			20.32; 1; <.001; .066			8.78; 1; .003; .138
** Perceived as difficult**	3101 (74.40)	300 (64.66)		173 (70.90)	127 (57.73)	
** Perceived as easy**	1067 (25.60)	164 (35.34)		71 (29.10)	93 (42.27)	
**Gender**			<.001°			.026°
** Man**	2076 (49.81)	206 (44.40)		112 (45.90)	94 (42.73)	
** Woman**	2083 (49.98)	233 (50.21)		112 (46.90)	121 (55.00)	
** Transman**	0	5 (1.08)		4 (1.64)	1 (.45)	
** Transwoman**	0	1 (0.22)		1 (.41)	0	
** Other**	9 (.22)	19 (4.09)		15 (6.15)	4 (1.82)	

Because the comparisons in this table involved 2 sets of 6 independent tests, we adopted a Bonferroni-corrected significance level of .05/6 = .008 for these two sets of analyses. Sociodemographic information presented for heterosexual participants and for participants who self-identified as LGB+ (LGB+), as well as for LGB+ who self-identified as being part of a minority group (Sexual Minority) and LGB+ that did not (Non-sexual Minority)

*Abbreviations*: *LGB+* Lesbian, gay, bisexual, pan−/omnisexual, asexual, other, *df* Degrees of freedom, V Cramer’s V, D Cohen’s d

* Independent sample t-test with equal variances not assumed (instead of chi-square-test): t; df; *p*-value; D

° Fisher’s Exact Test (instead of Chi Square Test): *p*-value

The comparison of the distribution of men and women across different age groups in the entire population aged 16 to 69 and those in our sample is presented in Table 2.

**Table 2:** Sample weights. A comparison in distribution between the Belgian population and the study’s sample

Age group	Sex at birth	Population N	Population proportion	Sample n	Sample proportion	Population/Sample
**16–24 years old**	Female	576,098	0.07	699	0.15	0.47
	Male	601,426	0.08	753	0.16	0.50
**25–49 years old**	Female	1,864,081	0.24	815	0.18	1.33
	Male	1,883,527	0.24	733	0.15	1.60
**50–69 years old**	Female	1,475,820	0.19	818	0.18	1.05
	Male	1,458,421	0.19	814	0.18	1.05
**Total**		7,859,373	1.00	4632	1.00	

### Sexual orientation

Table 3 shows an overview of the proportion of the self-identified sexual orientations in the total sample and per sex at birth. In total, 10.01% (*n* = 464) identified with a sexual orientation label other than ‘heterosexual’ and were thus classified as LGB+. Male and female participants were equally likely to self-identify as LGB+ (χ^2^ = 2.29; df = 1; *p* = 0.131; V = 0.022), but male participants identified more often as gay and female participants as bisexual or pan−/omnisexual (χ^2^ = 28.28; df = 1; *p* < 0.001; V = 0.267).

**Table 3:** The proportion of participants per sexual orientation, presented in total and per sex at birth

	Total (*n* = 4632) n (Valid %)	Male (*n* = 2300) n (Valid %)	Female (*n* = 2332) n (Valid %)
**Sexual orientation**			
** Heterosexual**	4168 (89.92)	2085 (90.65)	2083 (89.32)
** Bisexual**	179 (3.86)	70 (3.04)	109 (4.67)
** Gay/Lesbian**	129 (2.78)	87 (3.78)	42 (1.80)
** Pan−/Omnisexual**	88 (1.90)	34 (1.48)	54 (2.32)
** Asexual**	28 (0.60)	8 (0.35)	20 (0.86)
** Other**	40 (0.86)	16 (0.70)	24 (1.03)

The first column of the table contains the variables used and their different categories. The second column gives the distribution of the total sample across the different categories of each variable. The third column contains the distribution of individuals that were assigned the male sex at birth. The fourth column contains the distribution of individuals that were assigned the female sex at birth

### Minority identity

Among the LGB+ participants (*n* = 464), 67.03% (*n* = 311) indicated possessing at least one characteristic that made them member of a minority group in Belgium. In this group, 17.89% (*n* = 83) considered themselves to be a member of a cultural minority because of their skin color, ethnicity and/or religion/life philosophy, 53.45% (*n* = 248) indicated to belong to the group of sexual and gender minority people; 19.18% (*n* = 89) to a minority group because of another characteristic, and 19.61% (*n* = 91) indicated to belong to more than one of these three minority group.

From the total sample, 5.48% (*n* = 254) indicated belonging to a minority group because of their sexual orientation. Just over half of the LGB+ participants identified as belonging to a minority group because of their sexual orientation (52.59%, *n* = 244). When we select the LGB+ participants who indicated to belong to a minority group because of their sexual orientation, 63.31% (*n* = 157) said that this was important for their identity.

### Mental health, quality of life and well-being

Table 4 presents the comparison between the observed mental health, quality of life, and well-being in heterosexual and LGB+ participants as well as the comparison of these variables between those LGB+ participants who identify as sexual minority and those who do not. Because each set of comparisons involved 12 independent tests, we adopted a Bonferroni-corrected significance level of .05/12 = .004 for these analyses.

**Table 4:** Observed mental health, quality of life, and well-being

Variable	Within total sample (*n* = 4632)		t; df; *p*-value; G	Within LGB+ group (*n* = 464)		t; df; *p*-value; D
	Heterosexual (*n* = 4168; 89.98%)	LGB+ (*n* = 464; 10.02%)		Sexual Minority (*n* = 244; 52.59%)	Non-Sexual Minority (*n* = 220; 47.41%)	
	Mean (SD)	mean (SD)		mean (SD)	mean (SD)	
**Quality of life**^a^	4.08 (0.72)	3.79 (0.85)	7.19; 540; <.001*; .395	3.84 (0.84)	3.72 (0.85)	−1.53; 462; .127; .044
**Resilience**^b^	3.29 (0.74)	3.02 (0.78)	7.60; 4630; <.001; .433	2.96 (0.80)	3.08 (0.76)	1.57; 462; .117; .146
**Depression**^c^	4.83 (4.81)	7.97 (6.68)	−9.85; 518; <.001*; .751	8.41 (6.68)	7.48 (6.66)	−1.51; 462; .132; .140
**Anxiety**^d^	4.85 (4.46)	7.07 (5.47)	−8.42; 534; <.001*; .622	7.43 (5.36)	6.67 (5.57)	−1.49; 462; .136; .139
**PTSD**^e^	0.58 (1.22)	1.28 (1.73)	−8.41; 516; <.001*; .585	1.26 (1.78)	1.30 (1.68)	.23; 462; .817; .022
	n (%)	n (%)	X^2^; df; *p*-value; V	n (%)	n (%)	X^2^; df; *p*-value; V
**Number of trusted person**s			1.47; 1; .225; .018			17.00; 1; <.001; .191
** 0 to 3**	2374 (57.19)	279 (60.13)		125 (51.23)	154 (70.00)	
** 4 and more**	1777 (42.81)	185 (39.87)		119 (48.77)	66 (30.00)	
**Hazardous alcohol use**^f^			.03; 1; .871; .029			2.66; 1; .103; .076
** Yes**	1279 (37.88)	174 (37.50)		100 (40.98)	74 (33.64)	
** No**	2589 (62.12)	290 (62.50)		144 (59.02)	146 (66.36)	
**Sedative use**			19.41; 2; <.001; .065			.03; 2; .986; .008
** No**	2830 (67.90)	268 (57.76)		141 (57.79)	127 (57.73)	
** Lifetime**	561 (13.46)	81 (17.46)		42 (17.21)	39 (17.73)	
** Past 12-months**	777 (18.64)	115 (24.78)		61 (25.00)	54 (24.55)	
**Cannabis use**			37.79; 2; <.001; .090			3.87; 2; .145; .091
** No**	3149 (75.55)	301 (64.87)		152 (62.30)	149 (67.73)	
** Lifetime**	607 (14.56)	76 (16.38)		38 (15.57)	38 (17.27)	
** Past 12-months**	412 (9.88)	87 (18.75)		54 (22.13)	33 (15.00)	
**Illegal drug use**			41.73; 2; <.001; .095			8.64; 2; .013; .136
** No**	3923 (94.12)	402 (86.64)		206 (84.43)	196 (89.09)	
** Lifetime**	148 (3.55)	31 (6.68)		14 (5.74)	17 (7.73)	
** Past 12-months**	97 (2.33)	31 (6.68)		24 (9.84)	7 (3.18)	
**Suicide attempt**			113.12; 2; <.001; .156			5.05; 2; .080; .104
** No**	3948 (94.72)	380 (81.90)		194 (79.51)	186 (84.55)	
** Lifetime**	188 (4.51)	69 (14.87)		38 (15.57)	31 (14.09)	
** Past 12-months**	32 (.77)	15 (3.23)		12 (4.92)	3 (1.36)	
**Self-harm**			227.13; 2; <.001; .221			6.77; 2; .034; .121
** No**	3790 (90.93)	319 (68.75)		155 (63.52)	164 (74.55)	
** Lifetime**	280 (6.72)	87 (18.75)		52 (21.31)	35 (15.91)	
** Past 12-months**	98 (2.35)	58 (12.50)		37 (15.16)	21 (9.55)	

This table presents the observed mental health, quality of life and well-being of heterosexual participants and for participants who self-identified as LGB+ (LGB+), as well as for LGB+ who self-identified as being part of a sexual minority group (Sexual Minority) and those that did not (Non-Sexual Minority). A corrected p-level of .05/12 = .004 was used as the critical significance level for both sets of comparisons

*Abbreviations*: *LGB+* Lesbian, gay, bisexual, pan−/omnisexual, asexual, other, *PTSD* Post Traumatic Stress Disorder, *SD* Standard Deviation, *df* Degrees of freedom, G Hedges’ g, D Cohen’s d, V Cramer’s V

^a^Quality of life: 5-point-Likert item; 1 = very poor to 5 = very good

^b^Brief Resilience Scale (BRS): Low (0·00–2·99), Normal (3·00–4·30), High (4·31–5·00), from 0 to 5

^c^Patient Health Questionnaire-9 (PHQ-9): Mild (5–9), Moderate (10–14), Moderately severe (15–19), Severe (≥20), from 0 to 27

^d^General Anxiety Disorder-7 (GAD-7): Mild (5–9), Moderate (10–14), Severe (≥15), from 0 to 21

^e^Primary Care PTSD Screen for DSM-5 (PC-PTSD-5): Acute stress symptoms present (≥3), from 0 to 5

^f^Alcohol Use Disorder Identification Test Short version (AUDIT-C): Yes (≥4 for females, ≥5 for males)

* Equal variances not assumed: Welch t-test statistic

From these findings, we derive that LGB+ participants reported poorer mental health, poorer quality of life, and poorer well-being than heterosexual participants. LGB+ persons reported significantly less resilience, more symptoms of depression, anxiety, and post-traumatic stress disorder (PTSD), and more (illegal) drug use, self-harming behavior and suicide attempts. Yet, the only difference between these two groups with a medium effect size, concerns self-harming behavior. No significant difference between these two groups was found for hazardous alcohol use or reported number of trusted persons.

Within the LGB+ group, the difference in observed mental health, quality of life and well-being between those who identify as sexual minority and those who do not, appears less significant. A significant difference in proportions of number of trusted people was only found between identification as belonging to a sexual minority and those that did not identify as sexual minority (*p* < 0.001).

Within the sexual minority group, the difference in observed mental health, quality of life and well-being between those that find their sexual related characteristics important for their identity and those that do not, were not significant (*p* > 0.05). These results were not added to Table 4 as none of the variables came out to be significant.

Respondents who self-identified as belonging to the sexual minority group reported an average of 1.88 (SD = 0.41) on the OBS-S (with scores ranging from 1 to 5 and where higher scores indicate greater minority stress). None of the respondents scored higher than 3.20, which means that no one reported a high level of minority stress (OBS-S value > 4). More than half (56%) of the respondents in the sexual minority group reported a high level of community connectedness (value > 4). The average community connectedness in this group is of 3.76 (SD = 0.84).

Respondents who self-identified as belonging to the sexual minority group and find their sexual orientation characteristics important for their identity scored significantly (t = − 3.23; df = 235; *p* < 0.001) higher on the OBS-S (mean of 1.95, SD = 0.41) compared to sexual minority respondents that did not find these same characteristics key to their identity (mean of 1.77, SD = 0.40). There was no significant difference between these two groups concerning the level of community connectedness (t = − 0.75; df = 245; *p* = 0.454).

## Discussion

This study was the first to include sexual orientation-related questions in a Belgian national representative population study. Based on this sample, we estimate that 10.01% of the population in Belgium self-identifies as LGB+, which is a higher rate than the estimates from earlier non-representative samples ranging from three to 8 % in Flanders [5, 6].

The most frequently reported non-heterosexual sexual orientation was bisexual (3.86%) followed by gay or lesbian (2.78%), pan- or omnisexual (1.90%), asexual (0.60%). A small number of 0.86% indicated to identify with a sexual orientation that was not mentioned in our list. Male and female participants were equally likely to identify with an LGB+ sexual orientation, but the distribution over the different LGB+ identity labels varied. LGB+ men were more likely than women to identify as gay, whereas women tended to label themselves more often as bisexual, pan−/omnisexual, asexual, or other compared to men.

Among the LGB+ participants, 67.03% identified with any minority group. Interestingly, only a little over half of the LGB+ participants indicated that they considered themselves as belonging to a minority group because of their sexual orientation (i.e., as sexual minority). This means that about half of this population either did not identify as belong to a minority at all or they did so because of another characteristic (e.g., because of their ethnicity, skin color, disability, age, …). LGB+ participants who did identify as part of a minority group were significantly older than those not identifying as belonging to a minority group. This could be explained by older participants potentially having been more exposed to othering experiences confirming their minority identities, but more research is needed to confirm this. Further, almost one in five LGB+ participants (19.61%) indicated to belong to more than one minority group. Qualitative research is needed to explore the relationship between self-labelling as LGB+ and the specific aspects of their minority identity. Future studies on LGB+ populations and sexual minority groups should thoroughly pilot their questionnaires given that our findings show that depending on the questions about sexual orientation versus sexual minority status, different study samples self-select. Participants who identify as LGB+ (*n* = 464) appear to be a different subgroup than those who identified as sexual minority (*n* = 254). Our findings suggest that using ‘LGB+ persons’ and ‘sexual minority people’ as synonyms will yield different prevalence rates and may introduce bias in estimating the proportion of non-heterosexual members of Belgian society.

In line with our expectations and previous international studies, we found mental health outcomes to be significantly worse for LGB+ participants than for heterosexual participants, although effect sizes were mostly small, with the exception of a medium effect size found for self-harming behavior. Contrary to what we expected, we could not detect any difference between these two groups in social support, as indicated by the number of trusted people. Studies on mental health disparities in minority groups often link the reported poorer mental health to sociodemographic variables [17–19]. In our sample, no differences were found in educational level, occupational status, or financial situation between the heterosexual and LGB+ participants. As such, these sociodemographic variables do not explain the observed differences. These two compared groups did however show a difference in mean age: the LGB+ participants were significantly younger than the heterosexual participants. Younger persons tend to apply different defense mechanisms and coping strategies compared to older persons [54], but more research is needed to study a potential age effect.

From the minority stress literature, we expected that among the LGB+ group, those who indicate to belong to a minority group report more mental health problems than participants without a minority identity. However, our findings did not support this proposition. There was only one exception: LGB+ participants who did not identify as minority reported significantly fewer trusted persons than self-identified minority people. Based on these findings, we cautiously hypothesize that LGB+ persons who do not identify with a minority group may experience less social support.

Moreover, within the LGB+ persons who identified as sexual minority, we found no significant differences in terms of mental health and well-being between those who considered sexual orientation related characteristics central to their identity and those who did not.

Also in contrast with our expectations and the international literature, the sexual minority group did not show high levels of minority stress. They also reported high levels of community connectedness, which may be linked to the more tolerant LGB+ climate in Belgium compared to other countries in the world [38]. The majority however reported a maximum of three persons that they trusted, but this observation was not significantly different from the general population. Yet, it is important to consider here that we only asked questions related to minority stress to those LGB+ persons who indicated belonging to a minority group and as such, we selected a subgroup of the LGB+ persons in our sample. The outcomes of our study could have been different if we would have included the entire group of LGB+ participants.

Within this group however, we did find a significant difference in reported minority stress related to the importance attached to sexual orientation and gender related characteristics for one’s identity. This leads us to hypothesize that minority characteristics which are considered central to one’s core identity might elicit minority stress.

Our study does not allow to draw causal conclusions, but given the low reported minority stress in our sample, there is no evidence in our findings that it may explain the observed difference in mental health, quality of life, and well-being between heterosexual and LGB+ participants. It may however explain why we did not find a general significant difference within the LGB+ group between those who did and those who did not identify with a minority group.

### Limitations

Although we strived for a perfect representative sample of the Belgian population, we recognize that the sample used in this study might differ from the general Belgian population in terms of educational level and language distribution. However, overrepresentation of participants with higher levels of education in research is common [55–57]. Nevertheless, given the potential regional differences in LGB+ accepting climates and their impact on the mental health, quality of life, and well-being of LGB+ persons [16, 24, 32, 38], future Belgian studies would benefit from balancing the language distribution and if possible the regional distribution over the Flemish, Brussels, and Walloon Region. Further, educational level may also impact our findings regarding experienced minority stress. We expect higher educated people to be more surrounded by other higher educated people. Higher educational status is positively linked to tolerant attitudes towards LGB+ persons [58, 59].

In this paper, we limited the analysis to exploring findings in the LGB+ population based on one self-identification item. Based on this question, we cannot examine differences among LGB+ individuals who identify with other sexual orientations than those assessed in our survey. Further, sexual orientation can also be measured using multiple dimensions including self-labeling, sexual/romantic attraction and sexual partners and behavior. By applying multiple dimensions to identify LGB+ persons, more individuals with a non-heterosexual identity may have been considered. Large differences emerge when sexual orientation is measured via self-identification versus via sexual behavior or sexual attraction [3, 4]. Lesbian, gay and bisexual identity estimates increase by 70% when respondents’ sexual behavior is considered (in addition to identity) and they double when sexual attraction is considered as a criterion [3]. However, another part of the explanation may be linked to what Coffman et al. (2017) observed: LGB people seem less likely to disclose their identification with an LGB+ label in self-identification questions compared to disclosing the sex of their sexual partners or the persons to whom they feel sexually attracted to [60]. Future studies could thus best measure the multiple dimensions of sexual orientation to get a better understanding of the sociodemographic characteristics, minority statuses, and associated health outcomes inherent to the subgroups defined based on sexual attraction, sexual behavior and sexual orientation labeling.

With regard to minority stress, we cannot examine its occurrence in LGB+ respondents who did not indicate that they are part of a minority group. Further research should present the items measuring minority stress to all LGB+ individuals – and by extension to total samples for comparison - regardless of whether they self-identify as belonging to a minority group or not. People may be exposed to stigma, prejudice and discrimination and potentially experience minority stress without this necessarily being associated with identifying that one has characteristics that may distinguish them from the majority of people in a given context.

Future studies could also benefit from exploring the relationship between internalized stigma, the importance attributed to minority characteristics and mental health. It is possible that internalized homophobia triggers LGB+ persons to devaluate their sexual minority characteristics to reduce internal conflicts.

## Conclusion

LGB+ individuals constitute a substantial portion of the general population, and they report elevated levels of mental health problems compared to heterosexual participants. The current knowledge regarding factors explaining these elevated levels remains limited. The current study showed that identifying as belonging to a minority group because of characteristics related to one’s sexual orientation, experiencing this as important for one’s identity, a lack of social support, and experiencing minority stress were not related to the observed mental health disparities. However, it is important for health care professionals to be aware that LGB+ persons run elevated risks. Further, it remains important to recognize the heterogeneous nature of this population and the importance of being sensitive to nuanced differences in subgroups within LGB+ populations.

## Supplementary Information

**Additional file 1.**

## Data Availability

We are unable to make our data set publicly available for ethical reasons. This study involves sensitive human research participant data, which cannot be shared publicly. However, the corresponding author can be contacted for future data request purposes (may require data use agreements to be developed).

## Abbreviations

AUDIT-C: Alcohol Use Disorders Identification Test-Concise
BNR: Belgian National Register
BRS: Brief Resilience Scale
DSD: Differences or disorders of sex development
GAD-7: General Anxiety Disorder-7
LGB: Lesbian, gay, bisexual
LGB+: Lesbian, gay, bisexual, pansexual, omnisexual, queer, questioning, fluid, asexual and have other sexual orientations
OBS: Othering-Based Stress
OBS-S: Othering-Based Stress Scale
OECD: Organisation for Economic Co-operation and Development
PC-PTSD-5: Primary Care PTSD Screen for DSM-5
PHQ-9: 9-item Patient Health Questionnaire
PTSD: Posttraumatic Stress Disorder
QR: Quick Response
SD: Standard Deviation
UN-MENAMAIS: Understanding the Mechanisms, Nature, Magnitude and Impact of Sexual Violence in Belgium
VAD: Vlaamse Expertisecentrum voor Alcohol en andere Drugs
