# Supplementary material for: An assessment of the proportion of LGB+ persons in the Belgian population, their identification as sexual minority, mental health and experienced minority stress
Source: BMC Public Health. 2022 Sep 23;22:1807. doi: 10.1186/s12889-022-14198-2 (PMC9502943; doi:10.1186/s12889-022-14198-2)
Supplement: Supplementary file 1 — Additional file 1. [file 12889_2022_14198_MOESM1_ESM.docx]

# An Assessment of the Proportion of LGB+ Persons in the Belgian Population, their Identification as Sexual Minority, Mental Health and Experienced Minority Stress

**Additional file 1**

Othering-based stress scale (OBS-S)

**Scoring:** The othering-based stress scale is scored by averaging all the subscales. Subscales are scored by averaging all the items relevant to the subscale. The subscale ‘Community connectedness’ should be reversed before including into the total Othering-based stress score. Total OBS scores can range from 1 to 5, with higher scores indicating greater othering-based stress.

There are 2 versions of the OBS-S: one adapted for sexual and gender minorities; one for persons who belong to a minority group because of their ethnicity, skin colour, religion or philosophy of life

**Introduction:** ***‘****You have indicated to have one or more characteristics that may distinguish you from the majority of the people in Belgium. This may make you part of a group of people with common interests or characteristics which distinguish them from the more numerous majority of the population of which they form a part. How frequently occur the situations described below in your life?’*

**The OBS-S for sexual and gender minorities**

| Subscale | Item | Scoring |
| --- | --- | --- |
| Identity concealment | - I avoid telling people about certain things in my life that might imply that I am LGBTQIA+ - I avoid talking about my love life because I do not want others to know that I am LGBTQIA+ - I do not bring a date to social events because I do not want others to know that I am LGBTQIA+ | 1= never  2= rarely  3= sometimes  4= regularly  5= all the time |
| Micro-aggressions | - I am expected to educate non-LGBTQIA+ people about LGBTQIA+ issues - People have re-labelled my identity, or referred to me by a name / pronouns that are different from how I identify myself - I have been accused of being too defensive or politically correct when talking about LGBTQIA+ issues with someone who is not LGBTQIA+ | 1= never  2= rarely  3= sometimes  4= regularly  5= all the time |
| Rejection anticipation | - When I meet someone new, I worry that they secretly don’t like me because I am LGBTQIA+ - I brace myself to be treated disrespectfully because I am LGBTQIA+ - I expect that others will not accept me because I am LGBTQIA+ | 1= never  2= rarely  3= sometimes  4= regularly  5= all the time |
| Victimisation events | - I have been verbally harassed or called names because I am LGBTQIA+ - Others have threatened to harm me because I am LGBTQIA+ - I have been bullied by others because I am LGBTQIA+ - I have overheard people making negative remarks about LGBTQIA+ people - I felt unsafe in a group of people because I am LGBTQIA+ - I have felt discriminated against on the labour and/or housing market because I am LGBTQIA+ - I have been physically attacked because I am LGBTQIA+ - I have had my personal property purposefully damaged by others because I am LGBTQIA+ - I have received unwanted sexual attention or been asked inappropriate questions about my sexual life because I am LGBTQIA+ - I have endured unwanted sexual contact because I am LGBTQIA+ | 1= never  2= rarely  3= sometimes  4= regularly  5= all the time |
| To what extent do you agree with the following statements? | | |
| Internalized stigma | - If I was offered the chance to be someone who is not LGBTQIA+, I would accept the opportunity - I wish I wasn’t LGBTQIA+ - I envy people who are not LGBTQIA+ | 1=strongly disagree 2=disagree  3=neither disagree nor agree  4= agree  5= strongly agree |
| Community connectedness | - I feel that I could find information and pamphlets on LGBTQIA+ issues - I feel that I could find professional services for LGBTQIA+ issues if I needed to - I feel that I could find a public space that is supportive of LGBTQIA+ activities | 1=strongly disagree 2=disagree  3=neither disagree nor agree  4= agree  5= strongly agree |

**The OBS-S for persons who belong to a minority group because of their ethnicity, skin colour, religion or philosophy of life**

| Subscale | Item | Scoring |
| --- | --- | --- |
| Identity concealment | - I avoid telling people about certain things in my life that might imply that I have a different skin colour, cultural or religious background - I avoid talking about certain traditions in my life because I do not want others to know that I have a different skin colour, cultural or religious background - I do not wear traditional clothing or symbols to social events because I do not want others to know that I have a different skin colour, cultural or religious background | 1= never  2= rarely  3= sometimes  4= regularly  5= all the time |
| Micro-aggressions | - I am expected to explain or educate others about issues related to my skin colour, cultural or religious background - People have re-labelled my identity, or referred to me by a name / pronouns that are different from how I identify myself - I have been accused of being too defensive or politically correct when talking about issues related to my skin colour, cultural or religious background with someone who is not familiar with it | 1= never  2= rarely  3= sometimes  4= regularly  5= all the time |
| Rejection anticipation | - When I meet someone new, I worry that they secretly don’t like me because of my skin colour, cultural or religious background - I brace myself to be treated disrespectfully because of my skin colour, cultural or religious background - I expect that others will not accept me because of my skin colour, cultural or religious background | 1= never  2= rarely  3= sometimes  4= regularly  5= all the time |
| Victimisation events | - I have been verbally harassed or called names because of my skin colour, cultural or religious background - Others have threatened to harm me because of my skin colour, cultural or religious background - I have been bullied by others because of my skin colour, cultural or religious background - I have overheard people making negative remarks about people with another skin colour, cultural or religious background - I felt unsafe in a group of people because of my skin colour, cultural or religious background - I have felt discriminated against on the labour and/or housing market because of my skin colour, cultural or religious background - I have been physically attacked because of my skin colour, cultural or religious background - I have had my personal property purposefully damaged by others because of my skin colour, cultural or religious background - I have received unwanted sexual attention or been asked inappropriate questions about my sexual life because of my skin colour, cultural or religious background - I have endured unwanted sexual contact because of my skin colour, cultural or religious background | 1= never  2= rarely  3= sometimes  4= regularly  5= all the time |
| To what extent do you agree with the following statements? | | |
| Internalized stigma | - If I was offered the chance to be someone with another skin colour, cultural or religious background, I would accept the opportunity - I wish I had a different skin colour, cultural or religious background - I envy people who don’t have this skin colour, cultural or religious background | 1=strongly disagree 2=disagree  3=neither disagree nor agree  4= agree  5= strongly agree |
| Community connectedness | - I feel that I could find information and pamphlets on issues related to my skin colour, cultural or religious background - I feel that I could find professional services for issues related to my skin colour, cultural or religious background, if I needed to - I feel that I could find a public space that is supportive of activities for people with the same skin colour, cultural or religious background as me | 1=strongly disagree 2=disagree  3=neither disagree nor agree  4= agree  5= strongly agree |
